# Artificial intelligence and machine learning disciplines with the potential to improve the nanotoxicology and nanomedicine fields: a comprehensive review

**DOI:** 10.1007/s00204-023-03471-x

**Published:** 2023-03-07

**Authors:** Ajay Vikram Singh, Mansi Varma, Peter Laux, Sunil Choudhary, Ashok Kumar Datusalia, Neha Gupta, Andreas Luch, Anusha Gandhi, Pranav Kulkarni, Banashree Nath

**Affiliations:** 1 Department of Chemical and Product Safety, German Federal Institute for Risk Assessment (BfR), Max-Dohrn-Straße 8-10, 10589 Berlin, Germany; 2 Department of Regulatory Toxicology, National Institute of Pharmaceutical Education and Research (NIPER-Raebareli), Lucknow, 229001 India; 3 Department of Radiotherapy and Radiation Medicine, Institute of Medical Sciences, Banaras Hindu University, Varanasi, 221005 India; 4 Department of Radiation Oncology, Apex Hospital, Varanasi, 221005 India; 5 Elisabeth-Selbert-Gymnasium, Tübinger Str. 71, 70794 Filderstadt, Germany; 6 Seeta Nursing Home, Shivaji Nagar, Nashik, Maharashtra 422002 India; 7 Department of Obstetrics and Gynaecology, All India Institute of Medical Sciences, Raebareli, Uttar Pradesh 229405 India

**Keywords:** Artificial Intelligence (AI), Nanomedicine, Physiologically based pharmacokinetic (PBPK) models, Nanotoxicology, Adverse outcome pathway (AOP) analysis, Machine Learning (ML)

## Abstract

The use of nanomaterials in medicine depends largely on nanotoxicological evaluation in order to ensure safe application on living organisms. Artificial intelligence (AI) and machine learning (MI) can be used to analyze and interpret large amounts of data in the field of toxicology, such as data from toxicological databases and high-content image-based screening data. Physiologically based pharmacokinetic (PBPK) models and nano-quantitative structure–activity relationship (QSAR) models can be used to predict the behavior and toxic effects of nanomaterials, respectively. PBPK and Nano-QSAR are prominent ML tool for harmful event analysis that is used to understand the mechanisms by which chemical compounds can cause toxic effects, while toxicogenomics is the study of the genetic basis of toxic responses in living organisms. Despite the potential of these methods, there are still many challenges and uncertainties that need to be addressed in the field. In this review, we provide an overview of artificial intelligence (AI) and machine learning (ML) techniques in nanomedicine and nanotoxicology to better understand the potential toxic effects of these materials at the nanoscale.

## Introduction

**Fig. 1:**
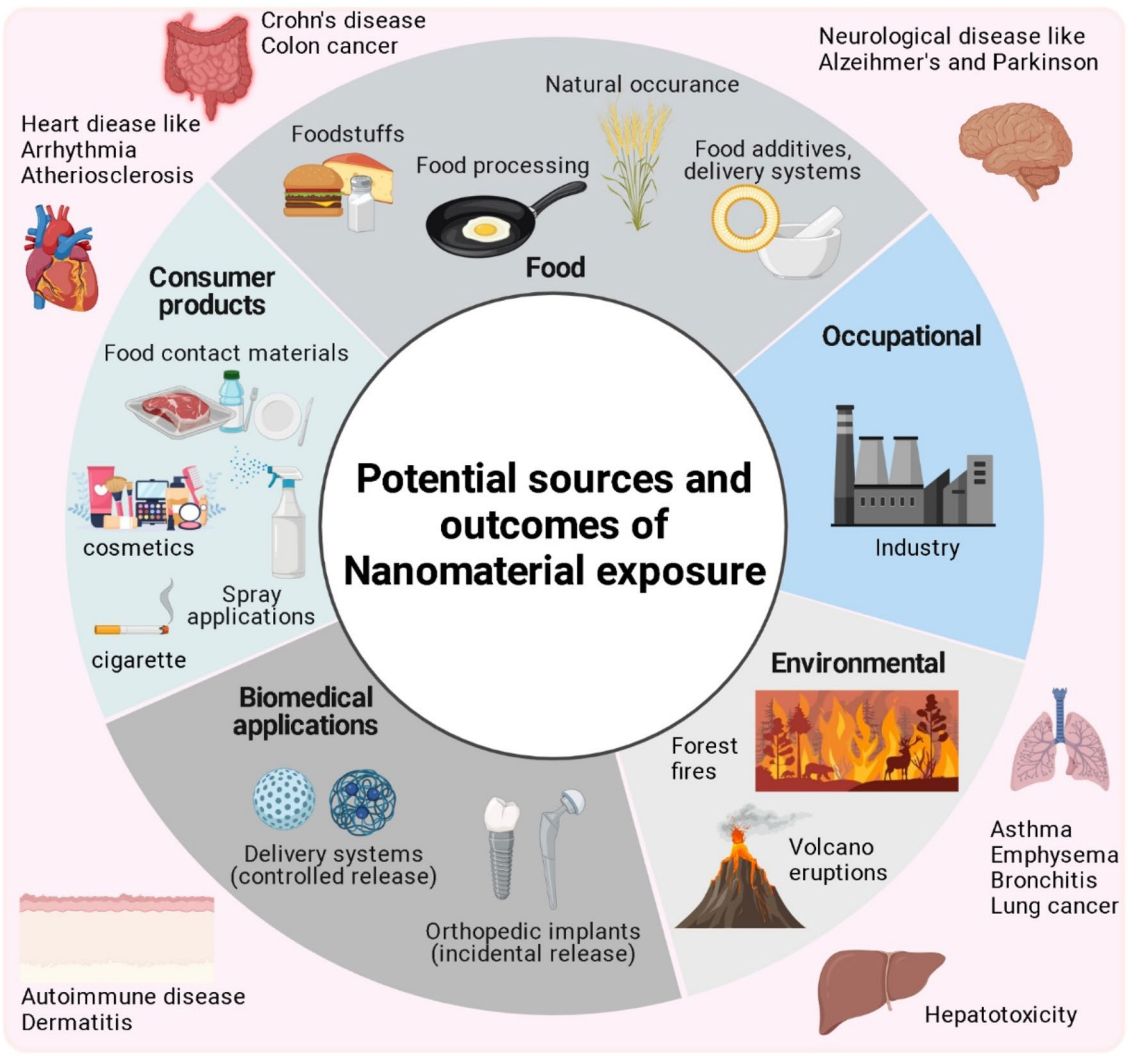
The potential sources (depicted in inner circle) and the health outcomes (depicted in outer square) of nanomaterial exposure (Ahamed 2014). This figure was made using Biorender.com

The nanomaterials are the most important advancement in science and technology worldwide. This invisible small size particles between 1 and 100 nm range have unique physical, chemical and biological properties which has applications in a wide range of fields (Bayda et al. 2019). According to a report by MarketsandMarkets, the global nanomaterials market is expected to reach $75.64 billion by 2025, growing at a compound annual growth rate of 13.2% from 2020 to 2025. Nanomaterials is widely used in everyday life such as sunscreen, cosmetics, food packaging, water filtration, medicine and energy production. Nanotechnology and nanomedicines have given world a wide range of benefits and will continue to do so but it has become highly necessary to address its undesirable effects. Nowadays, the world is facing huge number of diseases originating from the daily exposure of harmful chemicals or materials whose behavior is not known hiding behind their application and benefits (Domingues et al. 2022). We are exposed to nanomaterials through industries, food additives, processed food, cigarette, cosmetics, packaging materials, forest fires, controlled release medications, propellants, paints, etc. These have the potential to produce risk to human health and cause diseases like Parkinson, Alzheimer, asthma, cancer, emphysema, bronchitis, arrhythmia, dermatitis, vasculitis, urticaria, crohn’s disease, hypertension, thrombosis, podoconiosis and many more (Fig. 1) (Ahamed 2014; Asati et al. 2021). Hence, for a safer and healthier future, there is a need to address this issue and do efficient nanotoxicological testing. Since the market on nanoparticles is growing rapidly, there are huge number of nanoparticles created and are going to increase which is little difficult to regulate for its safety through in vivo and in vitro tests. With the high speed and volume data growing, it is becoming difficult to assess chemicals using traditional methods which is even more challenging to assess with various number of chemical toxicological endpoints (Singh et al. 2020a). Therefore, to meet the need to assess/predict the risk of such particles, we should opt for computational modeling methods that will save time and resources and help build community with better health.

## AI/ML modeling approaches for nanotoxicology related to systems biology and bioinformatics

**Fig. 2:**
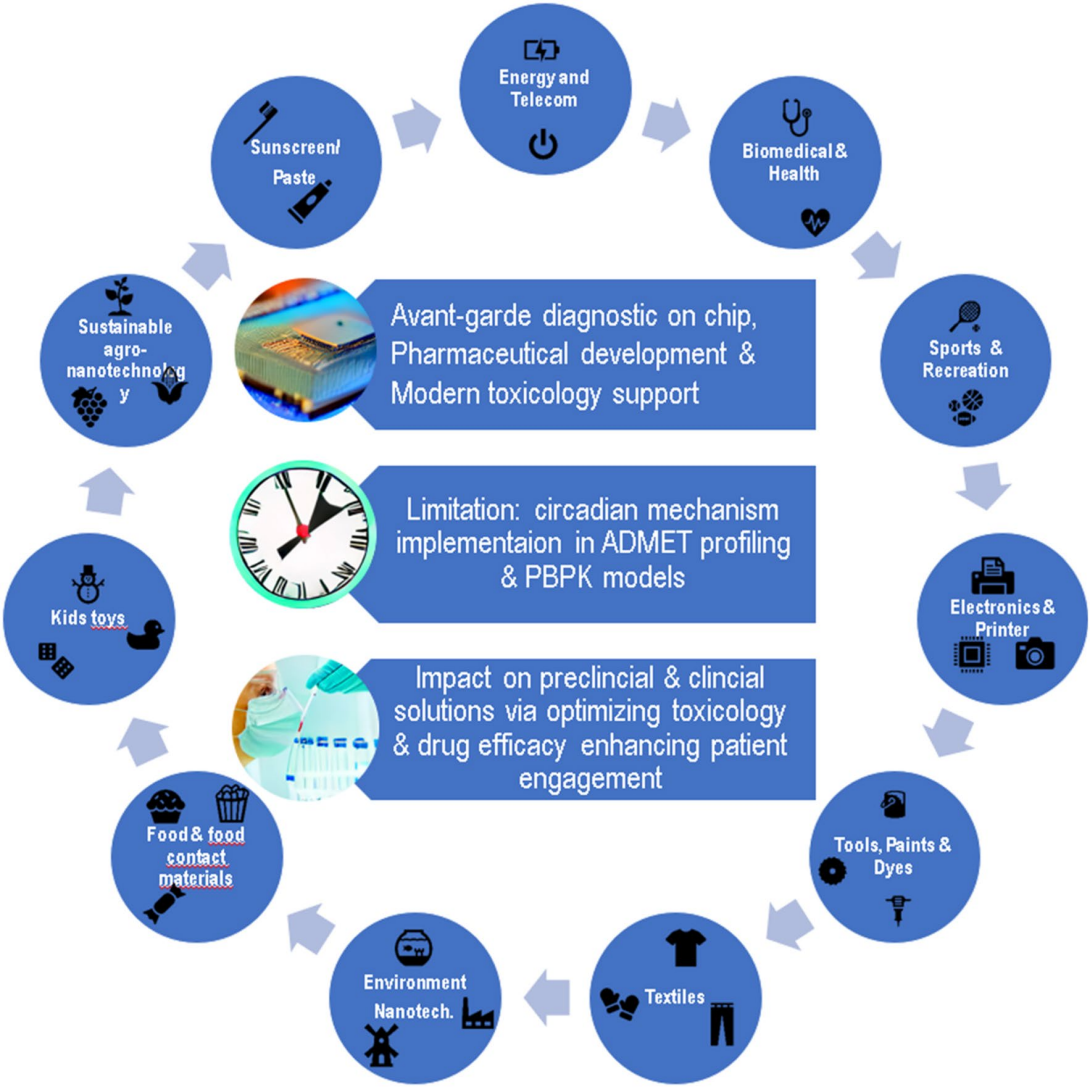
AI-ML application in wide sectors and their intrinsic worth in shaping human life

**Fig. 3:**
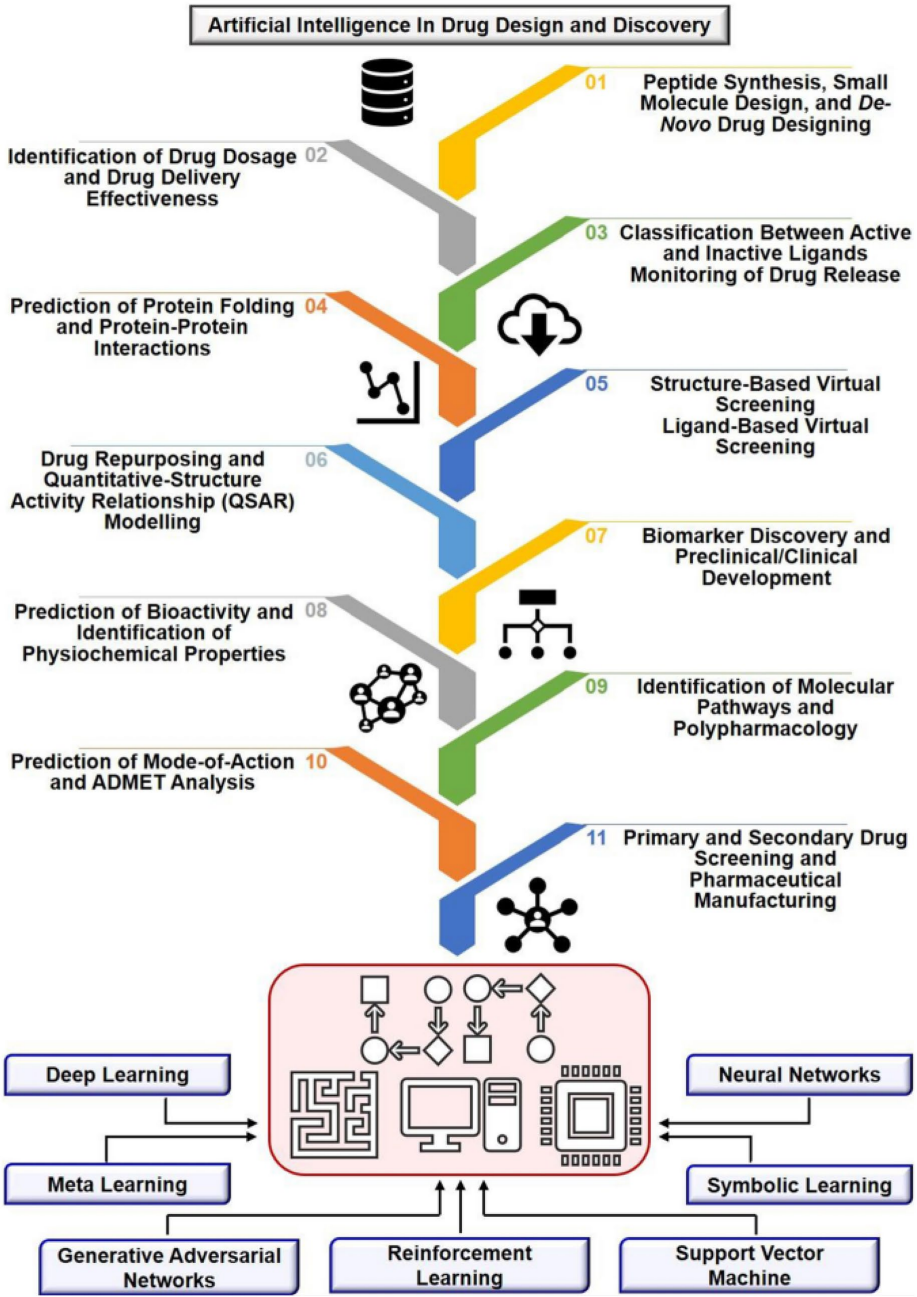
Artificial intelligence and machine learning in drug discovery and development (Gupta et al. 2021)

The science of artificial intelligence is evolving in revolutionizing way and has a significant impact on our lives. It has the potential to contribute significantly in number of fields including healthcare, finance, transportation, and manufacturing as well as resolving challenges like environmental protection, disaster response and social issues (Fig. 2). The AI is advancing and it has provided significant approaches to improve the process of drug discovery and development (Fig. 3). Research toward the field of systems biology and bioinformatics is focused toward the assessment of the adverse effects of the chemicals. Using bioinformatic tools and modeling, we can predict or can explain the adverse effects associated with any chemical.

AI enables to develop text mining and data mining approaches as a complement to more network biology and system biology approaches to understand mechanisms of diseases and the mechanisms of chemical actions (Kumar and Saha 2022). In this section, we explain briefly about shortcomings of chemical with respect to (w.r.t) consumer safety, currently in why we need approaches from the modeling field to understand that new chemicals entering in market can produce an adverse effect. More than 500 years ago as said by Paracelsus, “What is there that is not poison? All things are poison and nothing is without poison. Solely the dose determines that a thing is not a poison” who is in fact considered the father of toxicology and this is really key today and also current knowledge today that the importance of the dose of the compound to produce a toxic effect (Grandjean 2016).

Later, it was realized that it is not only the dose that is important but also the time dynamics and that is why it's important that we are able to apply different modeling approaches for understanding the mechanisms of chemical toxicity. As a background why researches are interested in chemical safety, as it is one of the major causes of attrition of new chemical during the whole process of chemical discovery and also after the chemical is marketed in context with regulatory affairs (Fuelle and Lanctin 2022). That is why the adverse reactions of chemicals are a very important concern in the clinical setting during the new area of application in consumer market and how can we predict these adverse chemical reactions? In general, the preclinical animal testing are failing to predict the human adverse chemical reactions, because they are only able to explain or predict this in 30% of the cases (Singh et al. 2021c). The main reason of that is because we still do not understand what are the mechanisms, why and how a chemical produces a chemical toxic and sub-toxic effect (e.g. genotoxicity, mutagenicity, etc.) and in fact for many chemicals which are there in the market, we do not have a detailed understanding of the mode of toxicological action and for asymptomatic adverse effect that is even worst (Singh et al. 2021b). Henceforth, it is important to realize that we are facing in our quest to understand the mechanisms of chemical toxicological mode of action and in particular chemical toxicity. We are addressing a multi-scale problem, because chemicals are in continuity exposed at organism level via different routes ca. dermal, ocular, inhalation and oral route (Chandrasekar et al. 2022). Subsequently, chemicals exert their action at the molecular level via chemical targets such as proteins, peptides, DNA, RNA or other molecules at the cell. Later, they translate the effect to tissues/organ level via specific cellular mechanisms (Singh et al. 2020b). Therefore, we need to cover all these scales at the functional level as well as at the temporal level to study the mode of action of tracks and also to understand the adverse effects.

## Quantitative systems toxicology

**Fig. 4:**
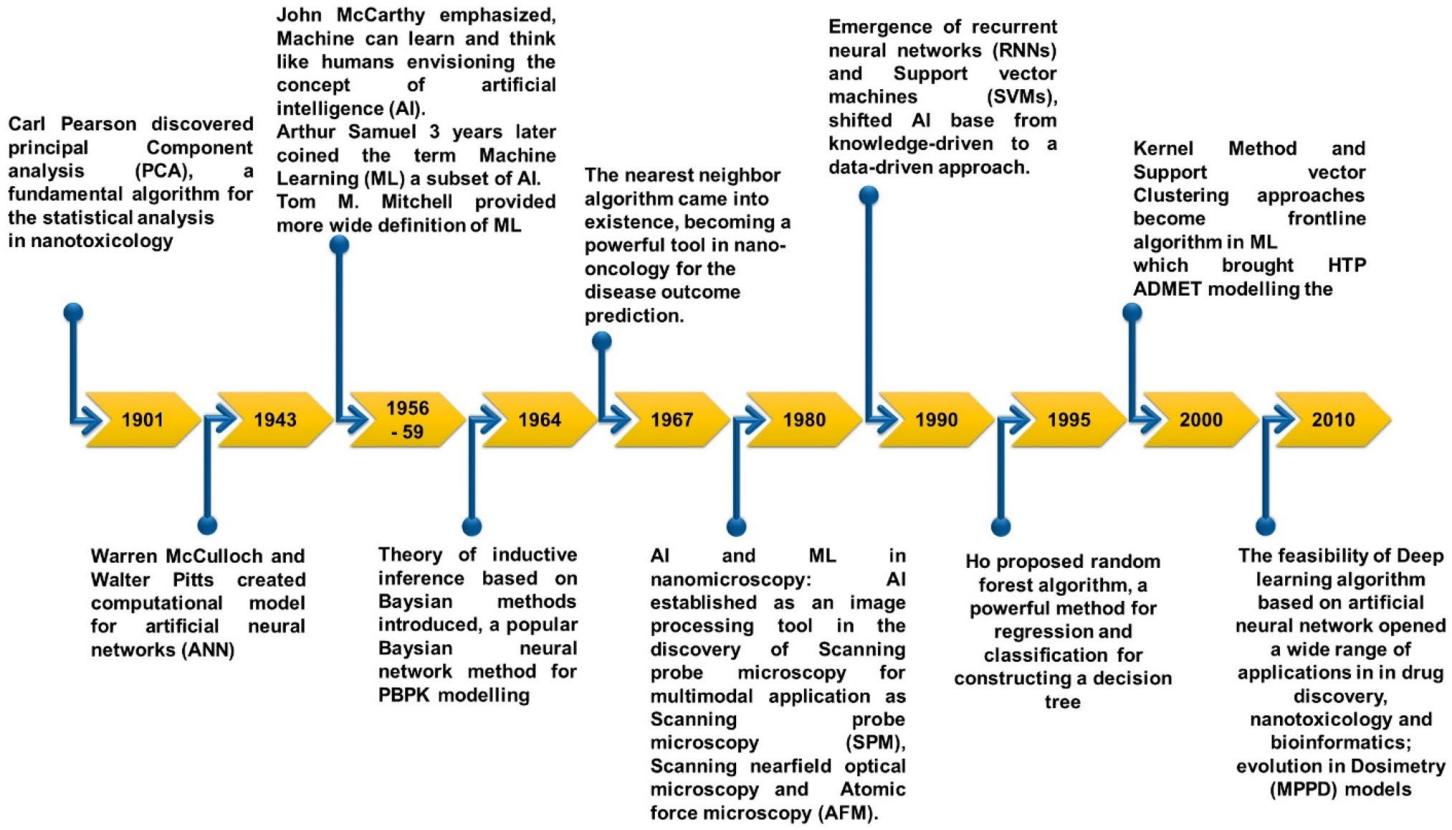
Development of nanomedicine & toxicology over time using AI and ML. Timeline for the evolution of AI, which includes statistical methods, as well as nanoparticles (NPs), commencing with the first synthesis and quantum effects discovered by Faraday in 1853. When AI was used for tasks like the identification of NP properties or interaction partners, the grouping of NPs based on their qualities or harmful effects, and the prediction of NP toxicity in 2010, both timeframes merged

Looking at history of toxicology, the QST started the testing of the toxic effect of chemicals and also chemical compounds in general in early in the last century when many people died from nephrotoxicity i.e. toxicity in the kidney due to the use of antibiotic (Petejova et al. 2019) (Fig. 4). Therefore, this incident prompted the testing of toxicity of compounds by using animal models and this is currently current practice in pharmaceutical industry before testing these compounds in humans and also from several legislation to regulate the toxicity of the compounds. A second very important event in the 1950s and 1960s was that of chemical Thalidomide which was used to relieve morning sickness and other types of symptoms in pregnant women produced teratogenic effects and more than 10,000 cases were reported (Kim and Scialli 2011). It is important to note that these toxic effects for Thalidomide could not be predicted by the animal studies that were conducted contemporarily in rat (Swaters et al. 2022). All these events highlighting it for a more systemic approach to do toxicology and the other facts that are have all these developments are what we already seen but is also current today that there is a poor translation from animal experiments to the human in vivo scenario. From that, it is obvious that not all the animal models are good models or good predictors to what clinicians will observe then in patients. That is why we need to address this and computational approaches are playing important role in modern toxicology (Hemmerich and Ecker 2020). Also, the cost is determining factor since there is a lot of resource investment to approve and develop new chemicals risk assessment and also another important issue is that currently a lot of animals are used for toxicity testing and there is a movement toward decreasing the use of animal testing in different types or different kinds of toxicity (3Rs principle) (Granath et al. 2014). This is the reason in the last decade, the field of quantitative systems toxicology emerged and this is the definition that we have that “the goal is to provide a quantitative understanding”. That is why we mentioned before the importance of those in time in the response of the toxic effect of a chemical and in the organisms for going from the molecular to the phenotypic observation. It is done by integration of computational approaches and different experimental methods. This is a broad approach that can be applied to a different kind of chemicals in particular can be applied to environmental toxicity testing better will focus today to their application for in the field of chemical risk assessment (Pérez Santín et al. 2021). In this area, it has some particular implications. In this section, we would like to summarize a little bit what are the mechanisms of or the key events of chemical actions and in how they produce adverse effects.

## Essential role of PBPK models and ADMET profilers in health hazard predictions

The chemical can have a desired effect by acting on various targets. It is important to note that some chemicals need to be metabolized into active compounds before they can act on the targets and produce the desired effect (Yu et al. 2018). There is also the potential for the chemical to produce other harmful effects. This can be directly caused by the action of the chemical or its metabolites on targets. Additionally, detoxification processes in the liver, such as the ADMET profile, convert the chemical metabolites into byproducts, which can be extracted from the body. These products can be chemically reactive and lead to DNA adducts modifications, mutations, and disruption of enzyme reactions. Many current QSAR models, molecular docking tools and ADMET properties prediction tool in silico are available to weigh the toxicity of chemicals (Daoud et al. 2021). Table 1 shows the comprehensive list of ADMET profiling tools, the parameters one can predict with their link adopted with permission from references (Shin et al. 2017). ADMET profilers and PBPK models are invaluable resources for connecting chemical toxicity and exposure data. They are essential for combining animal, in vitro, and computer-based experiments to aid in chemical assessments. Utilizing QSAR metabolic simulators, you can investigate if there are any known or simulated metabolites or hydrolysis products of the target chemical(s) (Yordanova et al. 2019).

**Table 1:** List of ADMET profiling tools (Shin et al. 2017)

Endpoints	Type	Program	Service
Absorption			
PAMPA	SW	MolCode toolbox	C
caco-2	SW	preADMET, MolCode toolbox, Discovery Studio, volsurf+, QikProp	W
	DB	the ADME database	W
MDCK	SW	preADMET, QikProp, ADMETpredictor	C
		preADMET	W
HIA	SW	ACD/percepta, preADMET, MolCode toolbox, Discovery Studio, ADMEWORKS Predictor, Stardrop	C
		PK/DB, admetSAR, preADMET	W
	DB	the ADME database, PK/DB	W
Skin permeability	SW	preADMET, ADMETpredictor	C
		preADMET	W
Corneal permeability	SW	ADMETpredictor	C
BA	SW	ACD/percepta, Impact-F	C
		PK/DB	W
	DB	Pact-F	C
		the ADME database, PK/DB	W
Distribution			
BBB	SW	ACD/percepta, preADMET, MolCode toolbox, Discovery Studio, ADMEWORKS Predictor, volsurf+, Stardrop, QikProp, ADMETpredictor	C
		PK/DB, admetSAR, preADMET	W
	DB	the ADME database, Chembench, PK/DB	W
VD	SW	ACD/percepta, volsurf+, ADMETpredictor	C
	DB	PK/DB	W
PPB	SW	ACD/percepta, preADMET, MolCode toolbox, Discovery Studio, volsurf+, stardrop, QikProp, ADMETpredictor	C
		PK/DB	W
	DB	Chembench, PK/DB	W
Partition coefficient	SW	MolCode toolbox, ADMETpredictor	C
Metabolism/excretion			
P-gP inhibition	SW	ACD/percepta, ADMETpredictor	C
		preADMET, PK/DB, admetSAR	W
	DB	the ADME database	W
P-gP substrates	SW	ADMEWORKS Predictor	C
		Chembench, admetSAR	W
	DB	Chembench	W
Regioselectivity phase 1	SW	QikProp	C
		FAME	W
Regioselectivity phase 2	SW	MEXAlert	C
		FAME	W
Phase II substrate/inhibitor	SW	preMetabo	W
Metabolic stability	SW	volsurf+	C

In this section, we will discuss the adverse effects of chemical metabolites, which include reactive products that can form adducts with other molecules. An example of this is conjugation with glutathione, which can lead to direct damage or deplete the cell's mechanisms of dealing with oxidative compounds that can then activate the body's regulatory response (Cooper and Hanigan 2018). If this response is activated in a homeostatic or small amount, the body can successfully cope with these reactive species. However, if it is continuously or highly activated, it can lead to cell and tissue damage. Additionally, this is the general overview of the action of chemicals on their targets. Traditionally, it was thought that chemicals act on their intended primary target by an effector pathway, leading to therapeutic effects (Yuan et al. 2018). Any off-target effects, which occur when the chemical acts on another target, activate a different effector pathway. However, recent research shows that this is not always the case (Cruz-Migoni et al. 2019). Understanding the mechanisms of toxicology is key to predicting the risk of adverse chemical reactions in patients. For example, when it comes to liver toxicity, it is important to be able to gauge the concentration of the chemical at the hepatocyte site at the liver cell, as well as measure different types of dynamic biomarkers such as transaminase in blood samples. Additionally, the understanding of the dynamics of enzymes in the liver can help to predict chemical-induced liver injury (Yu et al. 2018). When it comes to the heart, the action of chemicals on different ion channels in the cardiomyocyte can be assessed, and the dynamic biomarkers, such as changes in the acuity of the electrocardiogram or different types of depolarizations, can be used to predict arrhythmias. Combining different modeling approaches enables better predictions for chemical toxicity related with cardiac or lung anomalies via liver metabolic profiling of chemicals. For example, human ether-à-go-go-related gene (hERG) channel profiler is included in the toxicological categorization of many hERG QSAR models (Seierstad and Agrafiotis 2006). Such profiler are created using boundaries based on repeated dose toxicity test data pulled from the Hazard Evaluation Support System (HESS) database.

IPBPK models are at center stage to explore some of the current approaches for predicting the concentration of chemicals in different compartments of the body, such as the central components of PBPK models (Kuepfer et al. 2016). PBPK models are used to describe how chemicals are absorbed, distributed, metabolized and eliminated. Through the use of current or previous knowledge from literature, these models capture the underlying physiological and mechanistic components, with the ultimate goal of predicting the concentration of chemicals in the plasma and the site of action (Abouir et al. 2021). A PBPK model looks like a main compartment that consists of different blood circulation systems coupled to different compartments that represent the organs or tissues. Each compartment can be further specified and more information can be added in order to capture more mechanistic insight.

We can identify models associated to different types of toxicity, but the location of genes in these networks does not necessarily give us more insight into the mechanisms of regulation (Cordes et al. 2018). To gain a better understanding of the effector pathways, we need to incorporate additional data and use approaches such as linear programming algorithms or network-based approaches. We can also leverage on the wealth of omics data that has been generated for human and other organisms in order to construct the signaling layer. Mechanistic categorization schemes provide an organized way to identify key chemical properties based on published or expert knowledge (e.g. DNA binding by OASIS), helping to make informed decisions (Neuwoehner et al. 2008). Modeling chemical responses is of great importance, and the incorporating chemicals transformation into model has to be a key factor in metabolic modeling. In recent years, there has been a surge of research into genome-scale metabolic networks, which are used to simulate human metabolism at various levels, including cell, tissue, and organ. These networks are vast, with hundreds of coupled ordinary differential equations and thousands of metabolites and reactions, as seen in a model of the parasite and the comprehensive model of human metabolic (Carey et al. 2022). Developing these models requires immense effort, and often the collaboration of multiple institutions.

Therefore, how are metabolic models developed? This process starts by studying the enzymes and genes involved in metabolic reactions, using genomic, transcriptomic, and proteomic data to figure out if a particular protein is expressed in the tissue of interest (Wang and Zhang 2014). Then, all relevant literature is reviewed to identify the reactions taking place. After this information is represented in a set of equations, the model is simulated by constraint-based approaches to check if it matches the data. Since some of the reactions lack the necessary parameters, constraint-based approaches such as flux balance analysis are used to obtain quantitative analysis of metabolic flux at steady state (Dai and Locasale 2017).

## Expanding genome-scale metabolic network (GSMN) with structural information

How can we improve our metabolic model? Currently, there is a lot of research being done in this area. For example, adding information on protein structure and enzyme conformation can help us understand the impact of genetic variation. This includes knowledge on pharmacogenomics and genomics, which may influence toxic response of a chemical (Gu et al. 2019). Additionally, looking at the three-dimensional structure of proteins and incorporating information on sequence variations associated with chemical response and disease can help build a knowledge base. Finally, molecular dynamic simulations can be used to predict the effect of mutations in the protein structure (Singh et al. 2022b) and its function, particularly its binding to certain chemicals or drugs (Hirano and Kameda 2021). In this section, we will briefly discuss through the process of combining PBPK modeling approaches, genomic scale metabolic network, and a model that regulates gene expression of one of the key enzymes related to metabolism of a chemical. PBPK model can be used to predict the concentration of toxic metabolites in the liver in the presence of different perturbations, such as chronic stress in people exposed to certain toxic environment (Maldonado et al. 2017). Through this approach, one can calculate the chemical metabolized in the whole body while considering the metabolism in the liver and gene expression of key enzymes.

An advantage of QSAR and PBPK approaches is the ability to incorporate variability in chemical action into the models, though it is not clear how this is done (Knaak et al. 2012). However, open question remains that should compound toxicologists adjust the models according to individual patient data, or is the variability already included in the models as they are in current state. Structural information of a protein can be used to model the variation of its response to chemical use. This can be done by different approaches, such as building a population of models by changing different parameters (3D state of amino acids, alpha helices, beta sheets, etc.). Additionally, it is possible to include food interactions and stress in metabolism models by considering their effects at the gene regulation level (Yau and Potenza 2013). For example, an increase in cortisol impacts the synthesis of a particular enzyme, which is explained by a gene regulatory network (Simmonds et al. 1984).

## Nano-quantitative structure activity relationship (nano-QSAR)

There are different methods in computational modeling such as quantitative structure activity relationship (QSAR)/nano-QSAR, read across and data-driven profiling. The nano-QSAR or nano-QNTR (where N-nanostructure and T-toxicity) or nano-QNAR (where N-nanostructure and A-activity) approach can be useful in predicting the toxic potential of nanomaterials. The nano-QSAR approach statistically establish relationship between independent variables (physicochemical properties) and dependent variables (toxic effects) (Singh et al. 2023). In the past 2.1 decades, the research on QSAR and nanoparticles has grown tremendously especially after year 2012 showing the importance of computational modeling in field of nanotoxicology. To interpret the research trends investigation, four ways can be used, namely

- Cumulative curve
- Density visualization map of co-occurrence of keywords
- Thematic map
- Conceptual structure map and keyword clusters

**Fig. 5:**
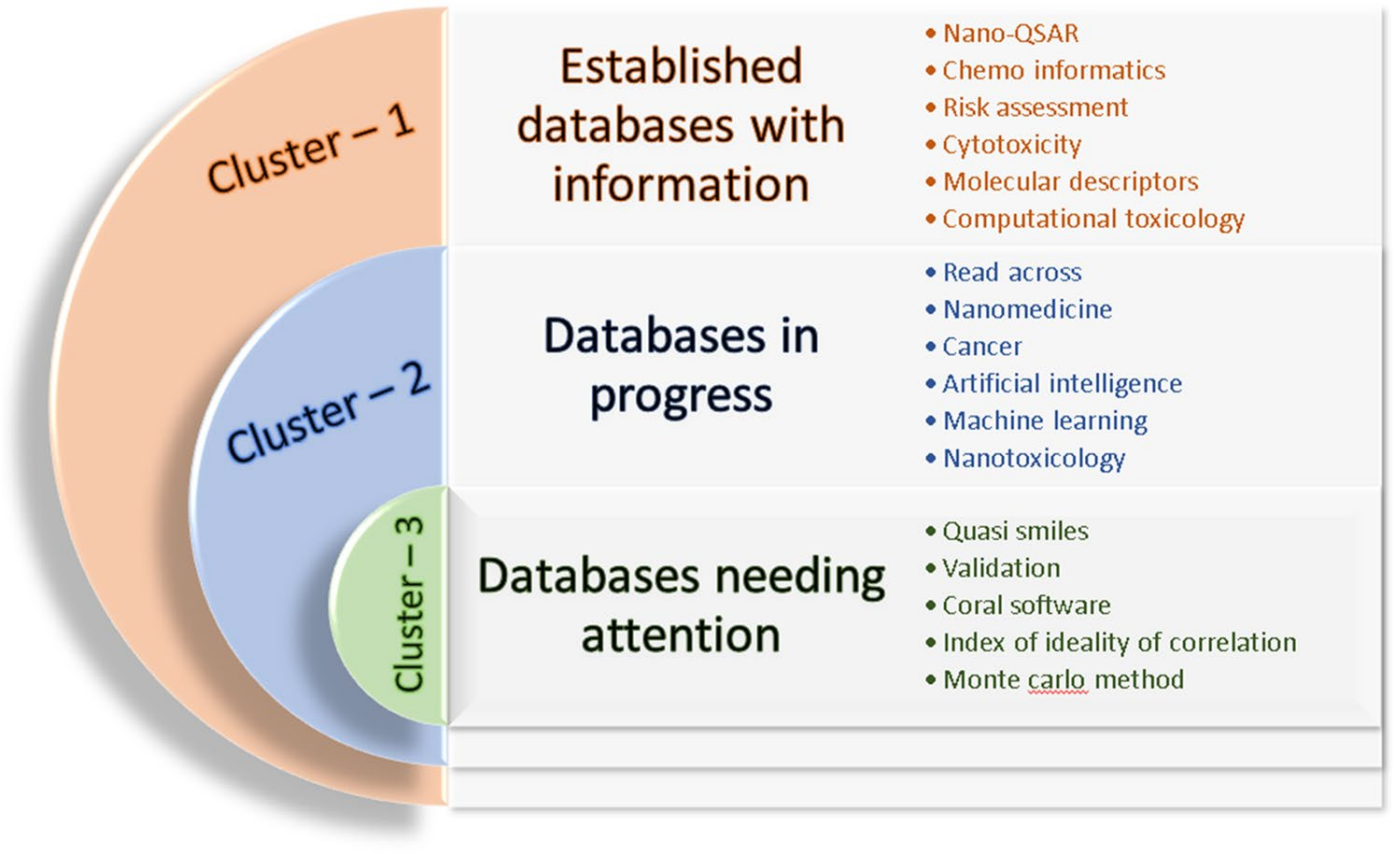
The conceptual structure map or bibliographic clustering analysis represented in three zones—red, blue and green cluster (color figure online)

The Density Visualization map involves a yellow–green–blue color scheme which reflects the hotspots of nano-QSAR research (Singh et al. 2019). These color schemes are in order of the decreasing item densities like yellow color represent hot research area (e.g.: QSAR, validation, cytotoxicity, etc.), whereas blue color represent the opposite. It is done via “VOS viewer” software (VOSviewer 2022). The Thematic map contains four quadrants namely motor theme, basic theme, emerging/declining theme, specialized/niche theme. The motor theme involve topics that have well-developed and important themes. It involves keywords like drug-delivery, descriptor selection and design (Di Cosmo et al. 2021). In basic theme, the topics important for research field but not developed are included, such as keywords optimal descriptors, cytotoxicity, prediction and toxicity. In Conceptual Structure map (Bibliographic clustering analysis), the themes are grouped into three clusters represented with red, blue and green colors including drug discovery; engineered nanomaterials; and correlation and logic (CORAL software), respectively. The clusters with large number of topics are considered to be saturated with research areas, while the less dense ones require more research or has scope for future (Fig. 5).

The nanoscale quantitative structure–activity relationship (nano-QSAR) is a computational technique which helps to understand the relationship between physical and chemical properties of nanomaterials and their biological effect on living organisms (Fortino et al. 2022). It predicts the biological activity of nanomaterials using quantum mechanics and statistical analysis. In nano-QSAR modeling, the mathematical relationship between variance in molecular properties (descriptors) and the variance in biological activity is obtained (Mikolajczyk et al. 2018). In nano-QSAR modeling, the primary objective is collection of data and data preprocessing.

The databases are obtained from various sources such as literature, databases, experiments and integrated sources. Under data collection, three main task comes into play—database, identify descriptors and endpoint selection (Singh et al. 2021a). The quality of data used determines the output of your assessment. Therefore, it is better to use various sources (good quality) rather than limited information on standardized protocol (poor quality) to get a reliable data output. The quality of nanoparticle data can be evaluated in several ways. One approach is to assess the accuracy and precision of the data, as well as the methods used to collect and analyze the data. Another approach is to evaluate the relevance and completeness of the data, and whether it is sufficient to answer the research questions at hand. Additionally, it is important to consider the credibility of the sources of the data and the expertise of the researchers involved in the study (Ballow et al. 1998). Ultimately, the quality of nanoparticle data can be determined by how well it meets the needs of the research and how well it stands up to scrutiny from other experts in the field. To evaluate the quality of obtained nanoparticle data referred as ‘Nanosecurity’, the criteria of “FAIR” principles should be fulfilled where “FAIR” stands for findable, accessible, interoperable and reusable, respectively (Ammar et al. 2020).

The typical QSAR/QSPR approach is assumed to be generated on complete, homogeneous data, which are obtained in the same conditions. Unfortunately, when nanomaterial characteristics are mostly partial or performed in varied conditions, it is hard to include this information in typical modeling. To overcome this issue, an approach called perturbation approach is used that merges different kind of experimental data independent of their measurement conditions by identifying the problem and then adding small variation term to predict solution. Hence, a combined nano-QSAR perturbation approach can help predict the toxicity of nanoparticles under different experimental conditions with better results (Wyrzykowska et al. 2019).

**Fig. 6:**
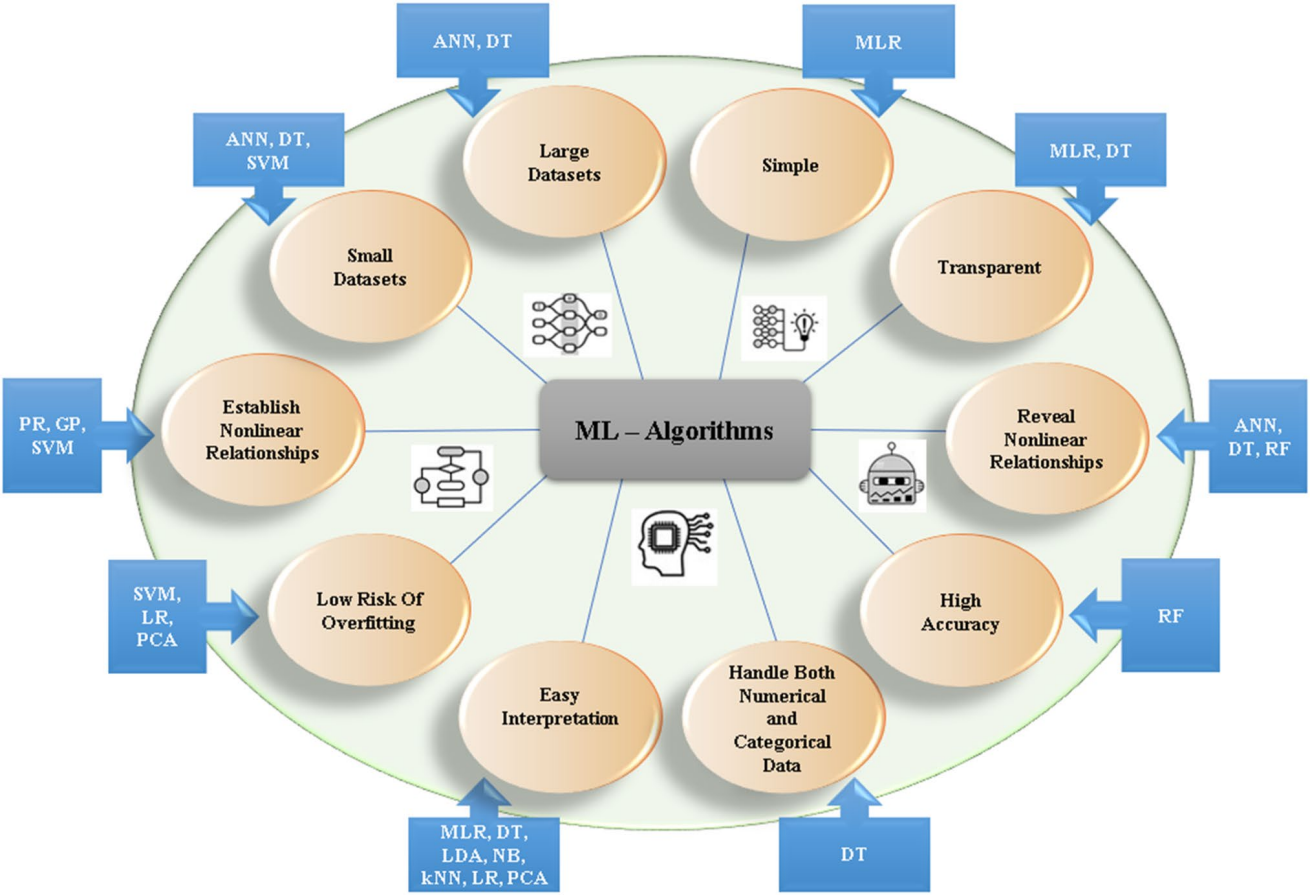
Advantages of different ML algorithms. For example, ANN is useful for both small and large datasets. SVM is suited for non-linear relationship and avoids overfitting. DT is versatile for transparency, empirical and categorical data. RF better fits with non-linear relationship with high accuracy

In a conventional nano-QSAR model, we could predict results with only one endpoint and, therefore, have to create multiple QSAR equations for each end points. What if we could include multiple endpoints in a single model equation? It can become more practical, robust, reliable and economical. This can be achievable using a multitarget or multitasking QSAR approach (mt-QSAR), the Box-Jenkins moving average method-based software “QSAR-Co” (QSAR-Co 2021) or an advanced python-based toolkit QSAR-Co-X (Ambure et al. 2019; Halder and Dias Soeiro Cordeiro 2021; QSAR-Co-X 2023). It uses a single QSAR model equation to predict end points with different experimental or theoretical conditions and different biological targets. As shown in Fig. 6, the upgraded version has advantages like high reproducibility of linear modeling; automatically perform the diagnosis of inter-collinearity among variables; reduced computation time by keeping only random division for dataset division; automatically generate validation set and calculate its statistical parameters; more number of Box-Jenkins operators; availability of Yc randomization to incorporate the influence of experimental elements; several non-linear modeling tools (kNN, SVM, RF, NB, GB and MLP); comparative analysis of multiple machine learning methods; hyperparameter tuning options for machine learning methods; and condition-wise prediction to understand how the developed model performs against individual experimental conditions, particularly for large datasets (Halder and Dias Soeiro Cordeiro 2021).

The applicability domain of a QSAR model is the response and chemical structure space in which the model makes predictions with a given reliability. According to OECD guidelines, applicability domain (AD) reflects the fact that QSARs are unavoidably associated with limitations in terms of the types of chemical structures, physicochemical properties, and mechanisms of action for which the models can generate reliable predictions (Maharjan et al. 2022). If a new compound exists in the AD of the developed model, only then can the developed model predict the compound precisely. It is extremely useful for QSAR developers to have information about the AD of the developed model to identify interpolation (true predictions) or extrapolation (less reliable predictions) (Veerasamy et al. 2011). The applicability domain can be defined using different methods such as the value range of the variables, value ranges of principal components of variables, optimal prediction space (TPKAT), geometric methods, probabilistic density distribution methods, distance-based methods, etc. The most common method is distance-based method (DM), defined as any numerical measure of the prediction uncertainty for a given compound by the model or measures the reliability of predictions. According to DM, the model is under AD if the distance from the molecule to the distance of the training set is lower than a predefined threshold. A new software to calculate AD is “Model disturbance index tool” available on NanoBRIDGES project website (NanoBridges; Ruiz and Gómez-Nieto 2018).

Model interpretation is a way to comprehend and provide an explanation for the variables that cause the model to produce a response function. There are two main approaches in model interpretation that is, machine learning (ML)-dependent and ML-agnostic. The ML-dependent model interpretation uses regression coefficients, rule extraction, layer-wise relevance propagation (LRP), CAM and GRAD-CAM. The ML-agnostic approach is based on sensitivity analysis, partial derivatives, feature importance by perturbation, integrated gradients and Shapley sampling values (Matveieva and Polishchuk 2021).

## High content image-based screening data and toxicological databases

**Fig. 7:**
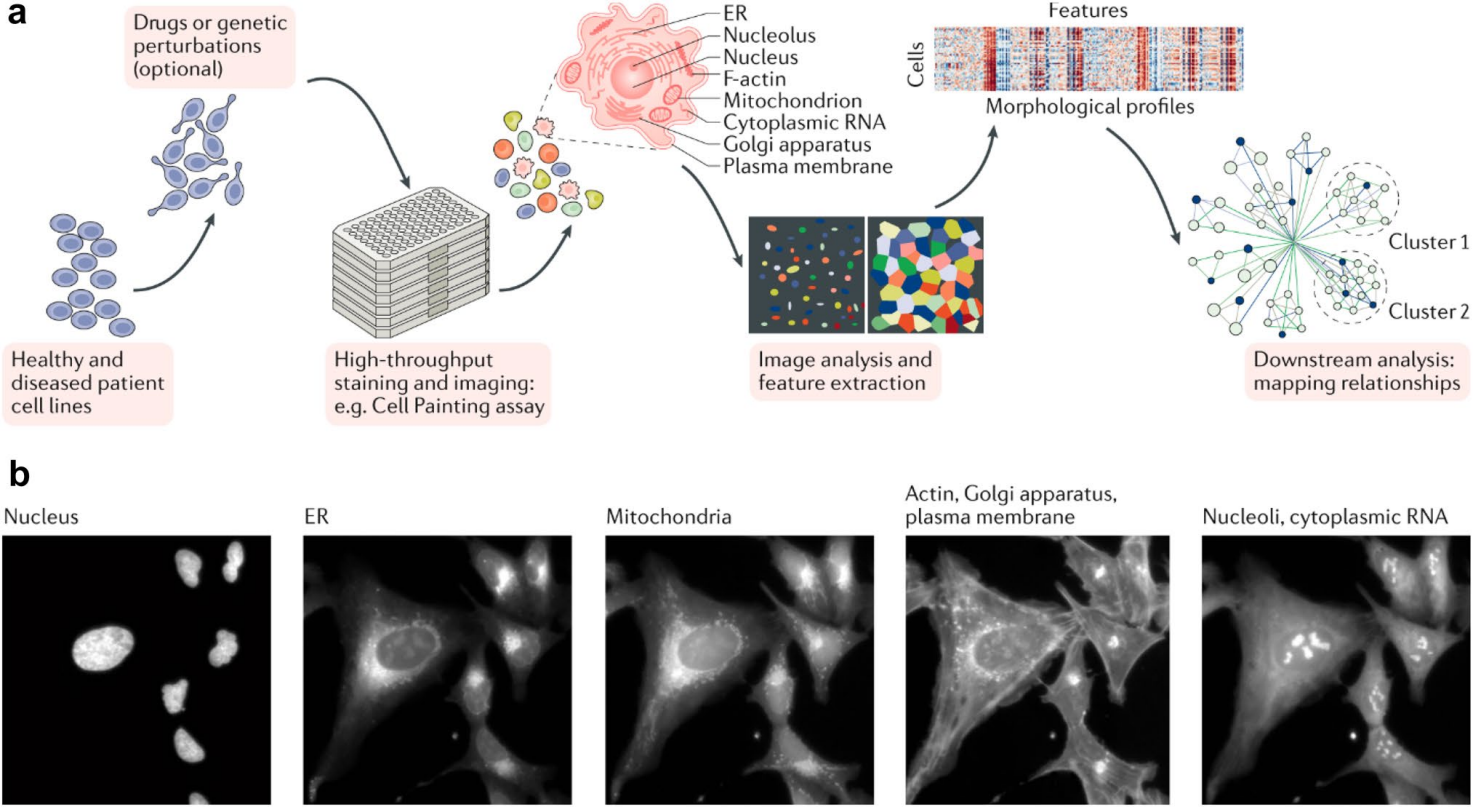
**a** An overview of typical steps in the workflow for generating image-based profiles from biological samples. **b** Example images from the Cell Painting assay often used for image-based profiling (Chandrasekaran et al. 2021)

The high-content image-based screening (HCIBS) is a type of phenotypic drug discovery approach where biological images are used to analyze cells and tissues specific compounds or information (Singh et al. 2022a). HCIBS data typically include images of cells or tissues, as well as quantitative data on the various cellular parameters being measured (Fig. 7). The data may be collected from cells or tissues that have been treated with different compounds or conditions, in order to identify changes in cellular phenotype or gene expression. HCIBS data can be used to inform the development of predictive models to improve the accuracy of toxicological predictions (Antoniou et al. 2019). This method involves automated imaging techniques that captures high-resolution images of the cells and these can be analyzed using image analysis software to extract quantitative data. This includes imaging technique such as fluorescence microscopy, confocal microscopy, atomic force microscopy, etc. (Chandrasekaran et al. 2021; Lin et al. 2020).

In today’s world of internet, there are many sources available to gather an information where data are huge and scattered. An effective platform is required to gather relevant data at one place and deliver an integrated accurate information. The big data can be characterized by attributes like high volume of data (high-content screening/HCS), speed of data generation (high-throughput screening/HTS), different types of data (omics, chemical structures), variability (genetic/population variations), validity (specific endpoint), visibility (access to data sources) and adequacy for specific goal. Although the data on toxicology is huge but its concern over sharing, accessibility, processing, quality, comparability, interoperability, integration and relevancy limits the applicability in predictive toxicology (Richarz 2019). It is possible to combine data from various sources and exposure from various media and chemical sectors (for example, collected within the framework of various legislations) at various times, and then integrate the data to provide an overall big data resource that is closer to the diverse real-life exposure to chemicals/chemical mixtures and for evaluating co-exposures. For a better evaluation of mixtures, data can be mined to identify trends and clarify the mechanisms underlying chemical interactions. There are many toxicological databases available online shown in the following table with their use and websites (Table 2) (Ji et al. 2021; Pawar et al. 2019).

**Table 2:** Different databases used in toxicology

Database	Usage	Website
Toxline (Toxnet)	Provide literature on biochemical, pharmacological, physiological, and toxicological effects of drugs and other chemicals	https://toxnet.nlm.nih.gov/
Aggregated Computational Toxicology Online Resource (AcToR)	Include chemical structure, physicochemical values, in vitro assay data and in vivo toxicology data	**–**
Gene-Tox database	Comprises mutagenicity data for more than 3,000 chemicals	https://www.nlm.nih.gov/databases/download/genetox.html
Toxicity Reference Database (ToxRefDB)	contains in vivo study data from over 5900 guideline or guideline-like studies for over 1100 chemicals	**–**
ChemBL	Brings together chemical, bioactivity and genomic data	https://www.ebi.ac.uk/chembl/target_report_card/CHEMBL245/
Toxicity Value Database (ToxValDB)	Includes data on thousands of chemicals with an emphasis on quantitative estimates of relevant points-of-departure from in vivo toxicology studies, such as no- and low-observable adverse effect levels, screening levels, reference doses, tolerable daily intake, etc	https://www.epa.gov/chemical-research/downloadable-computational-toxicology-data#:~:text=The%20Toxicity%20Value%20Database%20(ToxValDB,screening%20levels%2C%20reference%20doses%2C%20tolerable
RepDose and FeDTex database	Useful sources for No Observed (Adverse) Effect Level (NO(A)EL) or Lowest Observed (Adverse) Effect Level (LO(A)EL values from repeated dose studies for reproductive and developmental toxicity endpoints	http://cefic-lri.org/toolbox/fedtex/
HESS database	Information on 28 day repeat dose toxicity studies for 289 industrial chemicals in rats and includes additional rat metabolism datasets and information on ADME in rats and humans	https://www.nite.go.jp/en/chem/qsar/hess-e.html
ECOTOX	Provides information on adverse effects of single chemical stressors to ecologically relevant aquatic and terrestrial species	https://cfpub.epa.gov/ecotox/
T3DB (Toxin and Toxin Target database or Toxic Exposome database)	It combines detailed toxin data with comprehensive toxin target information	http://www.t3db.ca/
Comparative Toxicogenomics Database (CTD)	Includes more than 30.5 million toxicogenomic connections relating chemicals/drugs, genes/proteins, diseases, taxa, Gene Ontology (GO) annotations, pathways, and gene interaction modules	https://ctdbase.org/
Open-TG-GATES	Stores gene expression profiles and traditional toxicological data derived from in vivo (rat) and in vitro (primary rat hepatocytes, primary human hepatocytes) exposure to 170 compounds at multiple dosages and time points	https://dbarchive.biosciencedbc.jp/en/open-tggates/download.html
Toxygates	New interactive version of data from Open-TG-GATES covering 24,011 samples and 170 compounds	https://toxygates.nibiohn.go.jp/
ChemTunes ToxGPS	Consists of in vitro and in vivo toxicity endpoint specific alerting chemotypes; mechanism of action (MOA) based QSAR models, weight of evidence (WoE) outcomes, and ToxGPS datasets	https://chemtunes.com/
Nanoparticle Information Library	Share information about nanomaterials, including their health and safety-related properties	http://nanoparticlelibrary.net/
Cancer Nanotechnology Laboratory/caNano Lab	Expedite and validate the use of nanotechnology in biomedicine	https://cananolab.nci.nih.gov/
DaNa Knowledgebase	Help understanding the impacts of nanomaterials for humans and the environment	https://www.nanopartikel.info/en/
Nanomaterial registry	Help understanding the fundamental properties of nanomaterials	http://nanohub.org/
eNanomapper	Develop a computational framework for nano-toxicity data management	http://www.enanomapper.net/
S^2^NANO	Develop and commercialize safe and sustainable nano-products	http://portal.s2nano.org/
NanoSolveIT	A new concept, fingerprinting of nanomaterials, is proposed. Predicting the properties, functions, and hazards of nanomaterials by developing and integrating nanoinformatics	https://nanosolveit.eu/
EU NanoSafety Cluster	Study aspects include toxicology, ecotoxicology, exposure assessment, mechanisms of interaction, risk assessment, and standardization	https://www.nanosafetycluster/
NanoWerks Nanomaterial Database	Help the nanotechnology community to research nanomaterials	https://www.nanowerk.com/
Nanomaterial-Biological Interactions Knowledgebase	Help understanding the mechanism of nanomaterial exposure effects in biological systems	http://nbi.oregonstate.edu/
InterNano	Provides information about nanomanufacturing research, government reports on nanomanufacturing	http://www.internano.org/
NanoHUB	A web-based infrastructure for e-collaboration in the nanotechnology simulation community	https://nanohub.org/resources/
Nanotechnology Characterization Laboratory	Performs and standardizes the preclinical characterization of nanomaterials intended as cancer therapeutics	https://ncl.cancer.gov/
NanoMILE	Contain characterization data and high-throughput screening toxicity data of nanomaterials	https://ssl.biomax.de/nanomile/cgi/login_bioxm_portal.cgi
NanoDatabank	Design with simplicity of nanomaterial data storing and sharing	http://nanoinfo.org/nanodatabank/
PubVINAS	–	http://www.pubvinas.com/

## Challenges and future perspectives

Nano-QSAR modeling presents several challenges that can make it difficult to develop accurate and reliable models. Some of these challenges include:

- The complex and multi-dimensional nature of nanomaterials: The current list of descriptors is not enough to accurately predict the toxicity of nanoparticles due to high complexity and diversity of nanostructures. Thus, we need to find out some nano-specific descriptors that are most relevant to the activity of particular nanomaterial to get an accurate prediction of nano-toxicity.
- The lack of high-quality experimental data: In order to develop a reliable QSAR model, it is necessary to have a large and diverse dataset of experimental data. However, experimental data on the activity of nanomaterials are often limited, which can make it difficult to develop accurate models.
- The lack of standardization in the field: There are currently no widely accepted standards for describing the structures of nanomaterials, which can make it difficult to compare and evaluate different models.
- The potential for overfitting: Overfitting is a common problem in machine learning, where a model becomes too closely matched to the specific data used to train it, and thus performs poorly on new data. Overfitting can be a particular concern in nano-QSAR modeling, due to the limited availability of experimental data.
- The ability of nanoparticles to dynamically interact with the exposure conditions and trigger series of biological effect makes it even more difficult to predict the toxic potential of nanoparticle in different environment such as change in hydrophobic interactions, hydrogen bonding, corona formation with plasma and serum components.

However, there are some challenges specific to nano-QSAR modeling, including the complexity and variability of the structural properties of nanomaterials, as well as the lack of standardized methods for measuring their biological activity. As a result, the development of accurate and reliable nano-QSAR models can be a challenging task. To overcome these challenges, it is important to carefully select the training dataset and the descriptors used to represent the structural properties of the nanomaterials. The use of some new computational strategies to make diverse data more inclusive can help deal with shortage of homogenous experimental data. Additionally, the use of advanced machine learning algorithms and appropriate validation techniques can also improve the accuracy and reliability of nano-QSAR models.

## Data Availability

The data that support the findings of this review are available in the references cited in the text. Additional data related to this review are available from the corresponding author upon reasonable request.
